# Dieulafoy’s disease of the bronchus: report of a case and review of the literature

**DOI:** 10.1186/s13019-014-0191-8

**Published:** 2014-12-02

**Authors:** Yu Fang, Qingchen Wu, Bin Wang

**Affiliations:** Department of Thoracic & Cardiovascular Surgery, The First Affiliated Hospital of Chongqing Medical University, 1 Youyi Road, Chongqing, Yuzhong District, 400016 China

**Keywords:** Dieulafoy’s disease of bronchus, Hemoptysis, Embolization, Lobectomy, Bronchial artery

## Abstract

**Background:**

Dieulafoy’s disease is a vascular anomaly characterized by the presence of a tortuous dysplastic artery in the submucosa. Although frequently occurring in the gastrointestinal tract, multiple cases of Dieulafoy’s disease in the bronchus have been reported in the literature.

**Methods and results:**

We report a case of a 15-year-old boy suffering recurrent massive hemoptysis. Bilobectomy stopped bleeding after unsuccessful treatment with embolization of bronchial artery.

**Conclusion:**

It is concluded a congenital origin of this disease. Angiography and endobronchial ultrasonography can be used to diagnose Dieulafoy’s disease of bronchus whereas bronchoscopy biopsy should be avoided. Surgery is needed when embolization fails.

**Electronic supplementary material:**

The online version of this article (doi:10.1186/s13019-014-0191-8) contains supplementary material, which is available to authorized users.

## Background

Dieulafoy’s disease was initially described in 1898 by a French surgeon George Dieulafoy as a cause of bleeding in the stomach. It was defined as a vascular anomaly characterized by the presence of a tortuous dysplastic artery in the submucosa, from which vascular branches derive that can be located in the mucosa [1]. Apart from the stomach, this malformation has also been described in other organs of the alimentary tract, e.g., esophagus, duodenum, gall bladder, jejunum, colon and rectum [2]. In addition, a few cases of Dieulafoy’s disease of the bronchus have been reported. This lesion leads to massive hemoptysis which is a life-threatening condition associated with a high mortality rate. A prompt and efficient intervention is required in emergency to improve resuscitation and survival.

We present a case of a 13-year-old boy suffering from Dieulafoy’s disease of the bronchus without any previous respiratory disorders or history of tobacco use. When the published data are summarized, we focus on pathogenesis, pathology and clinical manifestations of the disease, and discuss strategies of diagnosis and treatment.

## Case presentation

**Figure 1:**
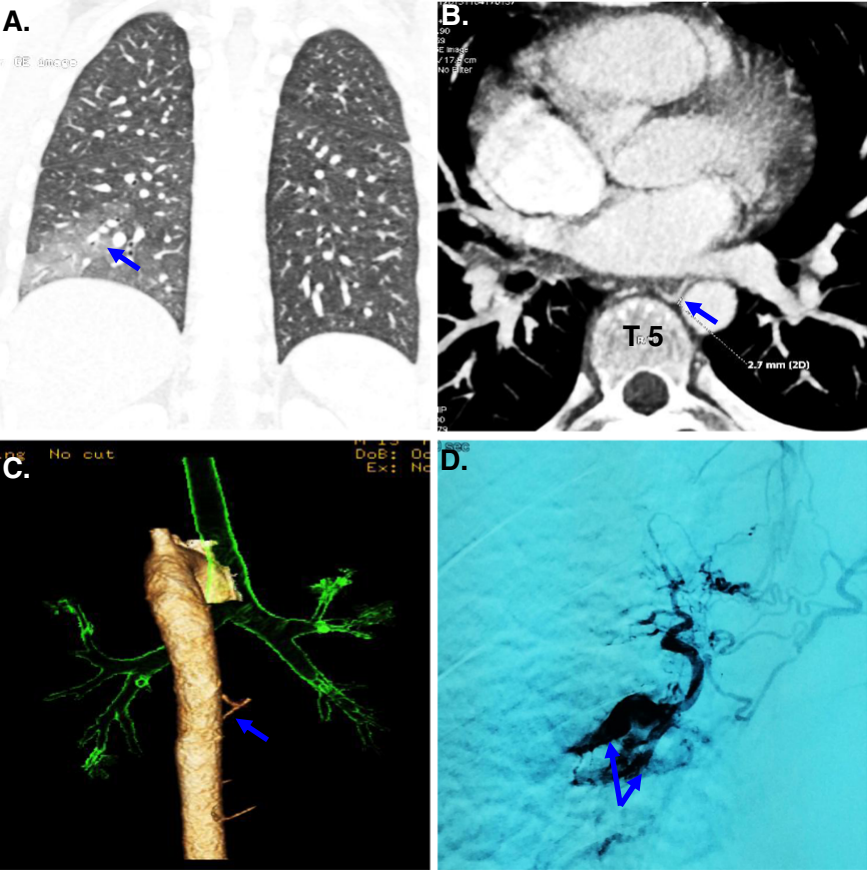
**Radiolographic findings of Dieulafoy’s disease of the bronchus. (A)** A chest CT showed exudative lesion, consolidation and atelectasis of the right lower lobe; **(B, C)** A CT angiography of bronchial artery showed a right bronchial artery arising from the anterior wall of the thoracic aorta at T5 level; **(D)** Angiography of the bronchial artery showed that a distal right branch of the bronchial artery arising from the thoracic aorta at T5 level was dilated and tortuous.

A 13-year-old boy was admitted to our hospital in emergency with massive hemoptysis. He had no history of any respiratory disorders or tobacco use. One week before admission, he coughed up approximately 500 ml of fresh blood and was admitted to another hospital. Chest CT scan showed exudative lesion, consolidation and atelectasis of right lower lobe (Figure 1A). He was immediately treated with Pituitrin and coagulation factors, and given red cell suspension and plasma. Hemoptysis was controlled temporarily.

**Figure 2:**
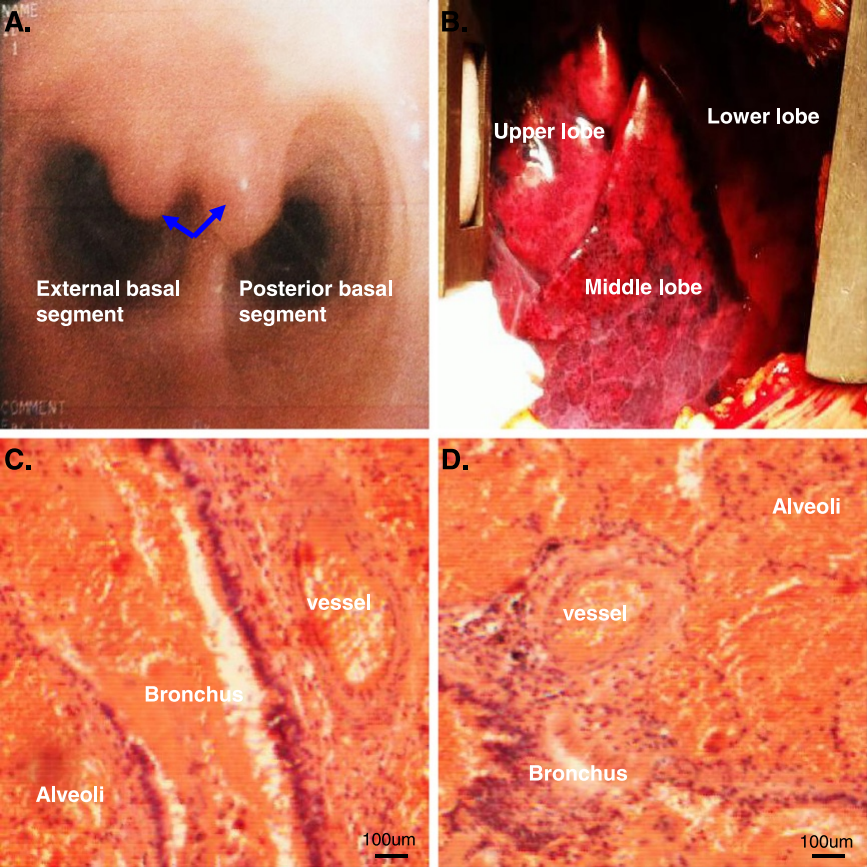
**Bronchoscopic and pathological findings of Dieulafoy’s disease of the bronchus. (A)** Bronchoscopic examination showed two polypoid nodules located at the carina between the external and posterior basal segment of the right lower lobe; **(B)** Macroscopic examination showed hemorrhage of the right middle and lower lobes; **(C** and **D)** Photomicrograph showing a dilated and tortuous bronchial artery branch close to the bronchus, infiltrating the entire bronchial wall and reaching the submucosa.

In order to find the cause of hemoptysis, he was transferred into our hospital for further diagnosis and treatment. Bronchoscopic examination showed two non-pulsating polypoid nodules of approximately 3-6 mm which are located at the carina between the external and posterior basal segment of the right lower lobe. The nodules were elevated about 1.5 mm above the surface and the covering mucosa appeared smooth (Figure 2A). Because of our suspicion of Dieulafoy’s disease of the bronchus, the endoscopist was refrained from performing biopsy and ordered endobronchial ultrasonography. Unfortunately, the ultrasound probe could not reach the lesion. A CT angiography of bronchial artery was performed and identified a right bronchial artery arising from the anterior side of the thoracic aorta at T5 level (Figure 1B, 1C). The tortuous, dilated and elongated branches of the bronchial artery coiled around right middle and lower bronchus. Bronchial artery angiography suggested a distal right branch of this bronchial artery was dilated and tortuous. Since this branch vessel was considered to be the cause of hemoptysis, arterial embolization was performed successfully (Figure 1D).

Three months after first admission, the patient was readmitted to our hospital in emergency with recurrent massive hemoptysis. Thoracotomy was performed. Obvious hematocele in the middle and lower lobe of right lung, especially in the middle lobe, was observed during thoracotomy. However intra-operative bronchoscopy failed clarifying the exact origin of hemorrhage. Consequently, a bilobectomy (the right middle and lower lobe) was successfully performed. Macroscopic examination showed hemorrhage of the right middle and lower lobe (Figure 2B). The lung parenchyma showed areas of hemorrhage and the bronchial lumens were filled with blood clots. Histologically, fresh bleeding and blood clots were seen in the bronchi, bronchioles and alveoli. Small vessels with diverse thickness opened directly into bronchial lumens (Figure 2C and D). After surgery, hemoptysis did not recur in the follow-up period of 5 months.

## Discussion

This is the first case of Dieulafoy’s disease of the bronchus in a teenager. Similar cases in the literature are summarized in Table 1[2]-[13]. Up to date, the etiology and pathogenesis of this disease remain uncertain. A large proportion of patients are non-smokers (11/17) and do not have any existing respiratory diseases (11/17). Three cases are teenagers or young adults, suggesting a possibility of congenital cause. It seems that gender or age is not associated with the disease, although the sample size is limited.

**Table 1:** **Clinical manifestations of Dieulafoy’s disease of bronchus** ^**1**^

Case	Age	Gender	Previous respiratory disease	Smoking history	Location	Treatment (Outcome)		Origin of vessels	Ref
						Embolization	Surgery		
1	32	M	no	no	RUL	failure	success	PA	[3]
2	42	M	yes	yes	RUL	success	-	BA	[4]
3	47	F	no	no	RML	-	success	BA	[5]
4	52	F	no	no	RLL	success	-	BA	[5]
5	35	F	no	no	RLL	failure	success	BA	[6]
6	59	M	no	yes	RML/RLL	-	success	BA	[6]
7	70	F	yes	yes	RUL	failure	-	PA	[7]
8	62	M	yes	-	RML	-	failure	BA	[8]
9	51	M	no	yes	RML/RLL	-	success	-	[2]
10	44	F	no	no	RML	success	-	BA	[9]
11	57	F	no	no	RUL/RML	success	-	BA	[10]
12	63	F	yes	no	RMB	-	-	-	[11]
13	28	F	no	no	LLL	-	-	-	[12]
14	41	M	no	no	RML	-	success	-	[13]
15	36	M	yes	no	RML	-	-	PA	[13]
16	61	M	-	-	RUL	-	-	-	[13]
17	15	M	no	no	RML/RLL	failure	success	BA	Our work

^1^Note: M: male, F: female; RUL: right upper lobe, RML: right middle lobe, RLL: right lower lobe; PA: pulmonary artery, BA: bronchial artery, RMB right main bronchus.

Several factors may cause hemoptysis in Dieulafoy’s disease of the bronchus. Iatrogenic injury (e.g., biopsy or lavage by bronchoscopy) is a confirmed cause leading to the rupture of lesion vessels and subsequent massive hemoptysis [5],[7],[8]. History of respiratory diseases [4],[7],[8],[11] (e.g., tuberculosis, bronchiectasis, chronic bronchitis and frequent pneumonia) and tobacco use [2],[4],[6],[7] may contribute to spontaneous fatal hemoptysis.

Normally, two or three branches of normal bronchial arteries usually less than 1.5 mm in diameter runs in parallel with the bronchi and creates a peribronchial plexus. The arterioles from this plexus with a diameter of <0.5 mm enter the bronchopulmonary segments, pass the muscular layer of bronchial wall and arrive beneath the bronchial submucosa [14]-[16]. When the diameter of an arteriole is larger than 2 mm, it is regarded as abnormal and may rupture [17]-[19]. In addition to bronchial artery, some branches arising directly from pulmonary artery may abnormally open to bronchial lumen. In this collection of 17 clinical cases, nine cases had arterioles arising from bronchial artery and three cases from pulmonary artery. These arterioles are approximately >2 mm in diameter, closely surrounding the bronchus and reaching the bronchial submucosa. It is believed that abnormal bronchial vessels are originated from abnormal development of the distal part of the sixth aortic arch, while abnormal pulmonary vessels are developed from the aorta, the upper aortic intercostal arteries or internal mammary arteries [2].

Dieulafoy’s disease of the bronchus presents non-typical symptoms, including a burst of cough, recurrent massive hemoptysis, chest discomfort and symptoms associated with low blood volume (e.g., dizziness, heart palpitation, cold clammy limbs, a constant decrease of blood pressure, etc.). Therefore, Dieulafoy’s disease of the bronchus should be considered if a patient suffering from frequent and recurrent hemoptysis and symptoms of low blood volume.

Angiography is a useful tool to disgnose Dieulafoy’s disease of the bronchus. Up to date, there is no specific diagnostic criterion to confirm a bronchial Dieulafoy’s lesion on angiography. Any abnormal arteries should be carefully evaluated. In particular, two kinds of abnormal blood vessels supplying the bronchus deserve attention: convoluted and elongated branches of bronchial artery and arterial plexus, which are primarily located on the right lobe or segment bronchus [4],[5],[9],[20], and new vessels arising from branches of pulmonary artery [12]. CT angiography is also a good tool to locate the origin of abnormal vessels and bleeding [9],[12].

Under the bronchoscope, endobronchial Dieulafoy’s lesion is characterized as small (usually <1 cm in diameter) and smooth elevated nodular lesions, often with white pointed caps and ridge-like bulge between nodules. The sessile nodules are usually covered with by normal-appearing bronchial mucosa and can be pulsating or non-pulsating [7],[8],[20],[21]. If Dieulafoy’s disease of the bronchus is misdiagnosed as benign submucosal lesions under bronchoscopy, biopsy may be taken and results in lethal bleeding. Therefore a bronchoscopist must exercise extra caution in case of hemoptysis and avoid biopsy when Dieulafoy’s disease is suspected. Even so, bronchoscopy is still a reliable way to examine endobronchial bleeding and, to some degree, to distinguish Dieulafoy’s lesion with malignant tumors and other benign lesions.

Up to date, there has been no consensus for treatment of Dieulafoy’s lesion of the bronchus. Treatment strategy needs to be based on disease presentation, site of the lesion and medical expertise available. Arterial embolization reduces the need for surgery and is obviously advantageous for patients who can not tolerate major surgery. Selective embolization of bronchial arteries were performed in this cohort, however, only half of them fully recovered [4],[5],[9],[20]. Several reasons may contribute to the failure of embolization of bronchial arteries: (1) abnormal vessels arise from pulmonary circulation rather than systemic circulation [7]; (2) Revascularization and neovascularization may cause recurrent hemoptysis after embolization [4]; (3) drop of the embolus dropping may cause of failure of an initially successful embolization. Therefore it is highly recommended to locate and follow up the bronchial lesions and their feeding vessels as precisely as possible with bronchoscopy in combination with angiography.

Surgical resection is the second option for hemoptysis caused by bronchial Dieulafoy’s lesion. It is indicated when the abnormal vessel arises from pulmonary circulation and when arterial embolization fails [3]. If malignant tumor is suspected as a possible reason of hemoptysis, surgical resection needs to be performed with a safe margin [2]. If recurrent hemoptysis takes place afterembolization, and arterial angiography suggests revascularization and neovascularization in the same lobe, lobectomy may be necessary [4].

## Conclusion

Dieulafoy’s disease of the bronchus may have a congenital origin, arising from either systemic circulation or pulmonary circulation. Considering minimal invasiveness, selective embolization of bronchial artery is considered the first-line treatment. If it fails, surgical resection will be needed. Arterial angiography is a safe and effective method for initial diagnosis and subsequent follow-up.

## Consent

Written informed consent was obtained from the patient for publication of this Case report and any accompanying images. A copy of the written consent is available for review by the Editor-in-Chief of this journal.

## Authors’ original submitted files for images

Below are the links to the authors’ original submitted files for images. Authors’ original file for figure 1 Authors’ original file for figure 2
