# Supplementary figures and images for: Dieulafoy’s disease of the bronchus: report of a case and review of the literature
Source: J Cardiothorac Surg. 2014 Dec 2;9:191. doi: 10.1186/s13019-014-0191-8 (PMC4263116; doi:10.1186/s13019-014-0191-8)

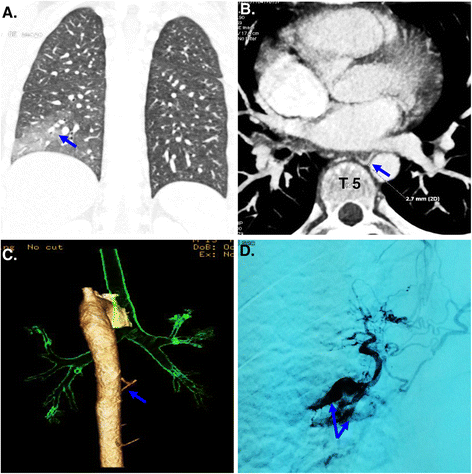

Supplement: Supplementary file 1 — Authors’ original file for figure 1 [file 13019_2014_191_MOESM1_ESM.gif]

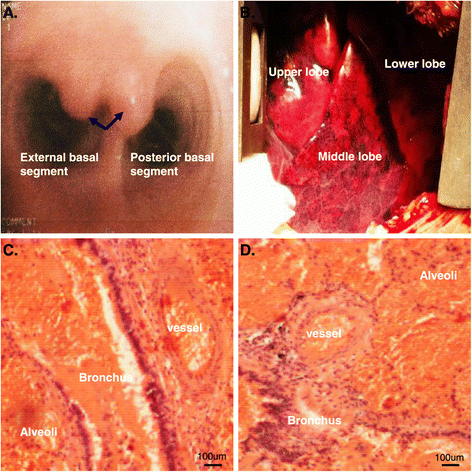

Supplement: Supplementary file 2 — Authors’ original file for figure 2 [file 13019_2014_191_MOESM2_ESM.gif]
